# Identification of Adipogenesis Subgroups and Immune Infiltration Characteristics in Diabetic Peripheral Neuropathy

**DOI:** 10.1155/2023/3673094

**Published:** 2023-01-19

**Authors:** Yumin Lin, Liyuan Qu, Jintao Wu, Meicen Pu, Yijuan Huang, Ying Cao

**Affiliations:** ^1^Department of Endocrinology and Metabolism, Nanfang Hospital, Southern Medical University, Guangzhou, China; ^2^Department of Orthopaedics, Ruijin Hospital, Shanghai Jiao Tong University School of Medicine, Shanghai, Shanghai, China

## Abstract

Dysregulation of adipogenesis is related to diabetic peripheral neuropathy (DPN) pathogenesis, which may be mediated by immune infiltration. Nevertheless, the expression patterns of multiple adipogenesis-related genes and the differences of immune infiltration in different lipid metabolism levels remain unknown. GSE95849, a gene expression matrix containing DPN patients and healthy participants, was downloaded from Gene Expression Omnibus (GEO) database. Differentially expressed adipogenesis-related genes (DEARGs) were screened by overlapping the adipogenesis-related genes with differentially expressed genes (DEGs). DPN patients from GSE24290 and GSE148059 were divided into two adipogenesis subgroups according to the expression of DEARGs. The single-sample gene set enrichment analysis (ssGSEA) was used to estimate the abundance of the immune cells between two subgroups. The analysis of immune infiltration suggested that a variety of immune cells and immune processes were elevated in the high expression group of DEARGs. The differentially expressed genes of the two subgroups were mainly enriched in biological processes and signaling pathways related to lipid metabolism. PPARG, FABP4, LIPE, FASN, SCD, DGAT2, PNPLA2, ADIPOQ, LEP, and CEBPA were identified as the hub genes of the two subgroups, whose related transcription factors (TFs) and miRNAs were predicted. An immunohistochemical assay was used to verify the expression of hub genes in DPN nerve tissues. Our comprehensive analysis of adipogenesis subgroups in DPN illustrated that different expression patterns of DEARGs may lead to different immune and inflammatory states. The identification of DEARGs may help to further distinguish the different characteristics of DPN patients and lay the foundation for targeted treatment. Our findings may bring a novel perspective to the diagnosis and treatment of DPN patients.

## 1. Introduction

Diabetic peripheral neuropathy (DPN) is a serious complication of diabetes characterized by pain and a loss of sensory function beginning distally in the lower extremities. Over time, at least 50% of individuals with diabetes eventually develop into DPN [1], which increases the risk of amputation and death. Nowadays, the exact pathogenesis of DPN is still unclear, and there is a lack of effective targeted treatment. There has been growing awareness that DPN is a chronic inflammatory disease, and this persistent chronic inflammatory response would accelerate the progression of nerve damage [2, 3], which is mainly related to oxidative stress, inflammation, and vascular endothelial system damage [4]. Related proinflammatory cytokines, such as TNF-*α*, interleukin- (IL-) 1, IL-6, and IL-8, are mainly produced by activated immune cells (especially resident macrophages) as well as adipocytes, which promote the amplification of inflammatory signals [5]. Exploring the role of immune infiltration and inflammatory responses may be crucial for further understanding the pathogenesis of DPN, which helps in searching for specific diagnostic markers and exploring potential effective therapeutic targets.

Adipogenesis is the process whereby fibroblast-like progenitor cells become triglyceride-filled mature adipocytes [6]. It can increase in size in one of two main ways: hypertrophy (increase in the size of existing adipocytes) or hyperplasia (formation of new adipocytes through differentiation of resident precursors known as preadipocytes) [6]. The subcutaneous adipose tissue (SAT) has a limited ability to expand by recruiting and/or differentiating available precursor cells. When inadequate, it will lead to a hypertrophic expansion of the cells with increased inflammation, insulin resistance, and dysfunctional prolipolytic tissue, leading to a disorder of lipid metabolism and lipid-related diseases [7–9]. Researches have shown that obesity was associated with an increased risk of incident DPN among individuals with type 2 diabetes mellitus, regardless of sex [10]. Emerging evidence showed that excessive adipogenesis was related to DPN pathogenesis [11, 12]. The excess free fatty acids catabolized by *β*-oxidation in response to hyperlipidemia can injure the peripheral nervous system, particularly Schwann cells, through the generation of ROS and mitochondrial dysfunction [13]. Multiple highly conserved pathways were reported to be associated with DPN, involving the signaling of adipogenesis, lipid synthesis, increased expression of adipogenesis-related factors, and inflammation [14, 15]. Adipogenesis is often accompanied by low-grade inflammation and immune response, secretion of adipokines, and release of fatty acids that can maintain immune cell activation [16]. Furthermore, obesity-associated inflammation can lead to complications in other metabolic tissues (e.g., liver, skeletal muscle, and pancreas) through lipotoxicity and inflammatory signaling networks [17]. Recent studies suggested that DPN was associated with an increase in inflammatory cells' aberrant cytokine expression, oxidative stress, ischemia, as well as proinflammatory changes in the bone marrow [18–20]. A large number of inflammatory mediators and cytokines (such as TNF-*α*, IL-1*β*, IL-18, IFN-*γ*, and IL-12) can promote the development of DPN. ROS generation, NF-*κ*B nuclear translocation, NLRP3 inflammasome (NLRP3, ASC, and caspase-1) activation, and gasdermin D cleavage may damage Schwann cells [21, 22]. Anti-inflammatory and antioxidant treatments can restore immune homeostasis and reduce pain caused by DPN [23, 24]. At present, numerous pieces of research have illustrated the effect of adipogenesis and inflammation on DPN and focused on the influence of a single gene on disease progression. However, according to the characteristics of the disease, the disease can be subdivided into groups, which will be helpful to carry out the targeted treatment. Besides, the expression patterns of multiple adipogenesis-related genes and the immune infiltration between different subgroups remain to be systematically analyzed.

In this study, we extracted differentially expressed adipogenesis-related genes (DEARGs) between DPN patients and healthy people, and two adipogenesis subgroups were discovered based on the expression of DEARGs. Then, we compared the immune infiltration and other biologic processes in two subgroups and selected the hub genes between them. Furthermore, transcription factors (TFs) and miRNAs that potentially regulate hub genes have been predicted. Immunohistochemistry was also utilized to verify the expression of hub genes. We expected that this study will make a certain contribution to the diagnosis and treatment of DPN.

## 2. Materials and Methods

### 2.1. Data Collection

All the microarray bulk RNA sequencing data we used were obtained from Gene Expression Omnibus (GEO) database (https://www.ncbi.nlm.nih.gov/geo/). GSE95849 (annotated by GPL22448 Phalanx Human lncRNA OneArray v1_mRNA platform) had 6 DPN patients and 6 healthy participants. The “limma” R package was used to normalize the expression matrix and analyzed differentially expressed genes (DEGs) between DPN and healthy people. GSE24290 (annotated by GPL10526 [HG-U133_Plus_2] platform) and GSE148059 (annotated by GPL16791 Illumina HiSeq 2500 platform) had 35 and 77 DPN patients, respectively. The “limma” package was used to remove their batch effect. Finally, 112 DPN patients from GSE24290 and GSE148059 were merged as a dataset for further analysis.

### 2.2. Identification of DEARGs

Adipogenesis-related genes of the HALLMARK_ADIPOGENESIS gene set were obtained from the MSigDB Team (https://www.gsea-msigdb.org/gsea/msigdb/), which is one of the most widely used and comprehensive databases of gene sets for performing gene set enrichment analysis. Log2|fold change (FC)| > 1 and adjust *p* value <0.05 were used as filter criteria to screen DEGs of GSE95849. Duplicated genes were excluded. Then, the intersection of adipogenesis-related genes and DEGs was analyzed by Venn diagram (FunRich 3.1.3), and 74 DEARGs were finally selected.

### 2.3. Unsupervised Clustering Analysis of DEARGs

The “ConsensusClusterPlus” R package was used to identify adipogenesis subgroups with an unsupervised clustering method according to the expression of DEARGs. The optimal value of *k* was chosen according to the following criteria: (i) a higher intragroup correlation and a lower intergroup correlation; (ii) the cumulative distribution function (CDF) curve increased smoothly while the delta area increased gradually; (iii) no subgroups have a small sample size. According to the above criteria, *k* = 2 was chosen as the appropriate number of clusters. Principal component analysis (PCA) was analyzed by the “states” R package. The “pheatmap” R package was used to visualize the expression of DEARGs in two adipogenesis subgroups.

### 2.4. Immune Infiltration in Two Subgroups

Chronic inflammation and immune infiltration were considered important processes which were induced by excessive adipogenesis [6]. To estimate the immune infiltration in two adipogenesis subgroups, single-sample gene set enrichment analysis (ssGSEA) was applied by “GSVA” and “GSEABase” R package [25, 26]. The “pheatmap” R package was used to visualize the expression of inflammation-related genes.

### 2.5. Gene Set Enrichment Analysis (GSEA)

GSEA 4.2.3 was used for gene set enrichment analysis. The gene sets associated with 50 well-defined biological states or processes were obtained from Hallmark gene sets in MSigDB. A *p* value <0.05 and false discovery rate (FDR) < 0.25 were deemed as significant difference between two subgroups.

### 2.6. Identification of DEGs in Adipogenesis Subgroups and Function Enrichment Analysis

To make a further understanding of the two subgroups, the “limma” package was performed to identify DEGs with |FC| > 1.5 and adjusted *p* value <0.05. Gene Ontology (GO) and Kyoto Encyclopedia of Genes and Genomes (KEGG) were performed by the “clusterProfiler” R package using a *q* value <0.05 as statistically significant enrichment.

### 2.7. Protein-Protein Interaction (PPI) and Hub Gene Selection

Search Tool for the Retrieval of Interacting Genes (STRING) database (https://cn.string-db.org/) and Cytoscape (version 3.9.1) were used to analyze the interaction of DEGs. In order to find the hub genes among DEGs, the “cytoHubba” plugin of Cytoscape was utilized, and the top ten genes were chosen.

### 2.8. Correlation Analysis between Hub Genes and Immune Infiltration

To assess the relationship between hub genes and immune infiltration, correlation analysis was utilized between 10 hub genes and 29 immune cells and processes. A *p* value <0.05 was deemed as a significant correlation.

### 2.9. Prediction of Transcription Factors (TFs) and miRNAs

ChEA3 is a web-based TF enrichment analysis tool that ranks TFs associated with user-submitted gene sets. The ChEA3 background database contains a collection of gene set libraries generated from multiple sources including ENCODE, ReMap, GTEx, Enrichr, and ARCHS4 databases [27]. FunRich (version 3.1.3) is a stand-alone software tool used mainly for functional enrichment and interaction network analysis of genes and proteins [28]. Based on these two softwares, we predicted the potential TFs and miRNAs that may regulate hub genes we found. The top ten predicted TFs were shown in the bar chart, and the predicted miRNAs were listed in table. Heat map was also performed to present the relationship between hub genes and TFs.

### 2.10. Specimen and Immunohistochemistry (IHC)

Formalin-fixed, paraffin-embedded sciatic nerve tissues were collected from 3 DPN mice and 3 healthy mice. Animal tissue samples were presented by Yaoming Xue research group of the Endocrinology Department of Nanfang Hospital. The sciatic nerve was taken from db/db mice and has been pathologically diagnosed as DPN. This study was approved by the Ethics Committee of Nanfang Hospital in Guangzhou. Primary antibodies used were as follows: monoclonal mouse anti−PPARG (60127-1-IG; 1 : 5000 dilution; Proteintech); monoclonal rabbit anti−FABP4 (ab92501; 1 : 16000 dilution; Abcam); polyclonal rabbit anti−LIPE (17333-1-AP; 1 : 200 dilution; Proteintech). The IHC assay was conducted as previously reported [29].

### 2.11. Statistical Analysis

R software (version 4.1.2) and Perl (version 5.16.2) were conducted to process, analyze and visualize data. Shapiro-Wilk test was applied for the normality test. The Student *t*-test was used for comparison of variables that obey normal distribution while the Wilcoxon rank-sum test was used for nonnormally distributed variables. A two-sided *p* value <0.05 was deemed statistically significant. The “ggpubr” and “ggplot2” R packages were applied for visualization.

## 3. Result

### 3.1. Identification of DEARGs

**Figure 1:**
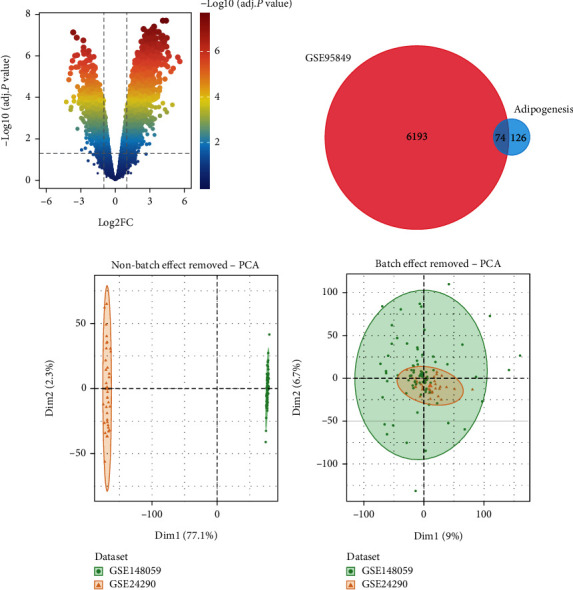
Identification of DEARGs. (a) Volcano plots of DEGs in GSE95849. Two vertical lines indicate gene expression fold change > 1 and <-1, respectively, and the horizontal line indicates the adjusted *p* value of 0.05. The color of the dot represents the adjusted *p* value levels. (b) DEARGs identified by Venn diagram. The blue circle represents the 200 adipogenesis-related genes from the MSigDB Team, and the red circle represents the 6193 DEGs in GSE95849. (c, d) PCA diagram indicated that after removing the batch effect, samples in two datasets became comparable.

The analysis process of this study can be seen in Figure S1. A microarray dataset, including 6 DPN patients and 6 healthy participants, was used to identify DEGs. A total of 6267 DEGs were obtained by the “limma” package, including 4463 upregulated and 1804 downregulated genes (Table S1). The final DEGs were visualized by the volcano map (Figure 1(a)). To determine the relationship between adipogenesis and DPN, we obtained 200 adipogenesis-related genes from the MSigDB Team. A total of 74 DEARGs were selected through the intersection of the DEGs and the adipogenesis-related genes (Table S2). The identified DEARGs were shown by the Venn diagram (Figure 1(b)).

### 3.2. Identification of Adipogenesis Subgroups in DPN

**Figure 2:**
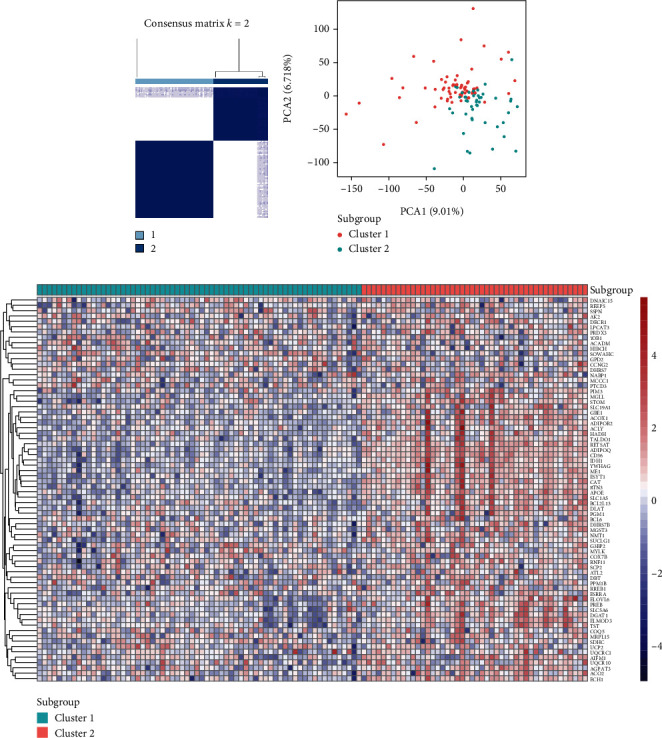
Identification of adipogenesis subgroups in DPN. (a) Consensus matrix heat map defining two clusters (*k* = 2) and their correlation area. (b) PCA analysis indicating an obvious difference in transcriptomes between the two subgroups. (c) Differences in expression levels of DEARGs between the two distinct subgroups.

GSE24290 and GSE148059 were obtained from the GEO database. After merging these two datasets, the “limma” R package was applied to remove the batch effect and normalized the gene expression matrix (Figure S2A-B, Table S3). PCA diagram indicated that after removing the batch effect, samples in two datasets became comparable (Figures 1(c) and 1(d)). 112 DPN patients were enrolled in this study to reveal the relationship between adipogenesis and DPN. Unsupervised clustering was used to identify adipogenesis subgroups based on expression of DEARGs. Our findings indicated that the optimal clustering variable was 2 (Figure S3A-I). As shown in Figure 2(a), we categorized DPN patients into two clusters with a reasonable number of patients in each cluster (*n* = 66, 46 in cluster 1 and cluster 2, respectively). PCA analysis indicated that cluster 1 and cluster 2 were distinguished into two parts as shown in Figure 2(b). Additionally, as displayed in Figure 2(c), a substantial difference in DEARGs expression was identified, most of which were upregulated in cluster 2.

### 3.3. Immune Infiltration in Adipogenesis Subgroups

**Figure 3:**
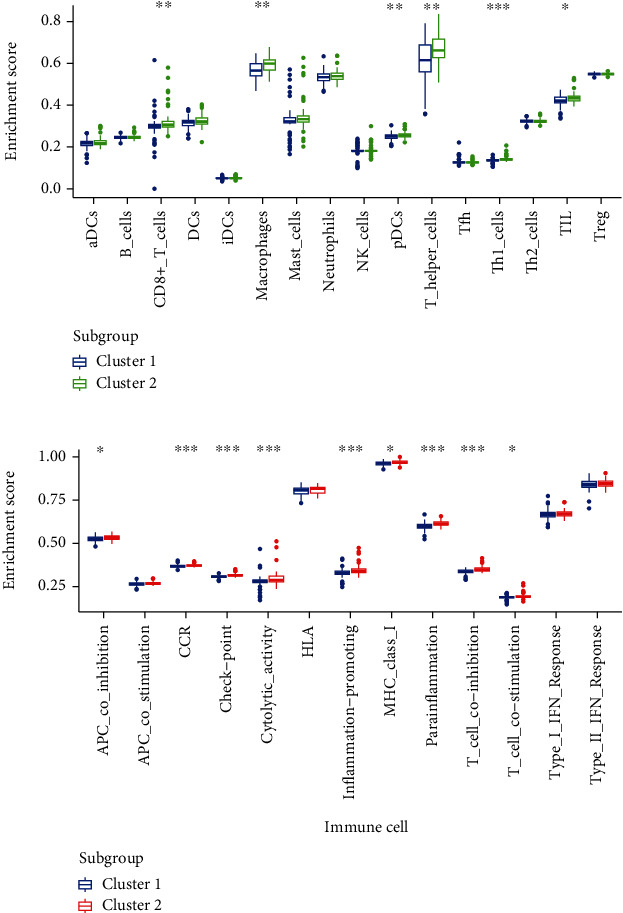
Immune infiltration in adipogenesis subgroups. (a) Abundance of 16 infiltrating immune cells in the two adipogenesis subgroups. (b) Differences of 13 infiltrating immune processes in the two adipogenesis subgroups. (^∗^*p* < 0.05, ^∗∗^*p* < 0.01, and ^∗∗∗^*p* < 0.001).

Immune infiltration and low-grade inflammation are both hallmarks of DPN [30]. We first compared the relative abundance of immune cells in two adipogenesis subgroups (Table S4). As shown in Figure 3(a), a significant difference in most immune cells between both clusters can be seen. The enrichment scores of CD8+ T cell, macrophage cell, pDCs, T-helper cell, Th1 cell, and TIL were significantly higher in cluster 2, in which there were higher-expressed DEARGs. We also assessed immune processes between two subgroups, which indicated that the enrichment scores of APC coinhibition, CCR, checkpoint, cytolytic activity, inflammation-promoting, parainflammation, T cell coinhibition, and T cell costimulation were markedly higher in cluster 2 than cluster 1(Figure 3(b)). Moreover, the expression of inflammatory-related genes was evaluated in two subgroups. Results showed that cluster 2 may have a more pronounced inflammatory response (Figure S4), which was consistent with previous mechanism research [16]. Chronic inflammation and immune infiltration were considered important processes which were induced by excessive adipogenesis [6]. This may prompt that the upregulated adipose differentiation process in DPN promoted immune infiltration.

### 3.4. GSEA Analysis in Adipogenesis Subgroups

**Figure 4:**
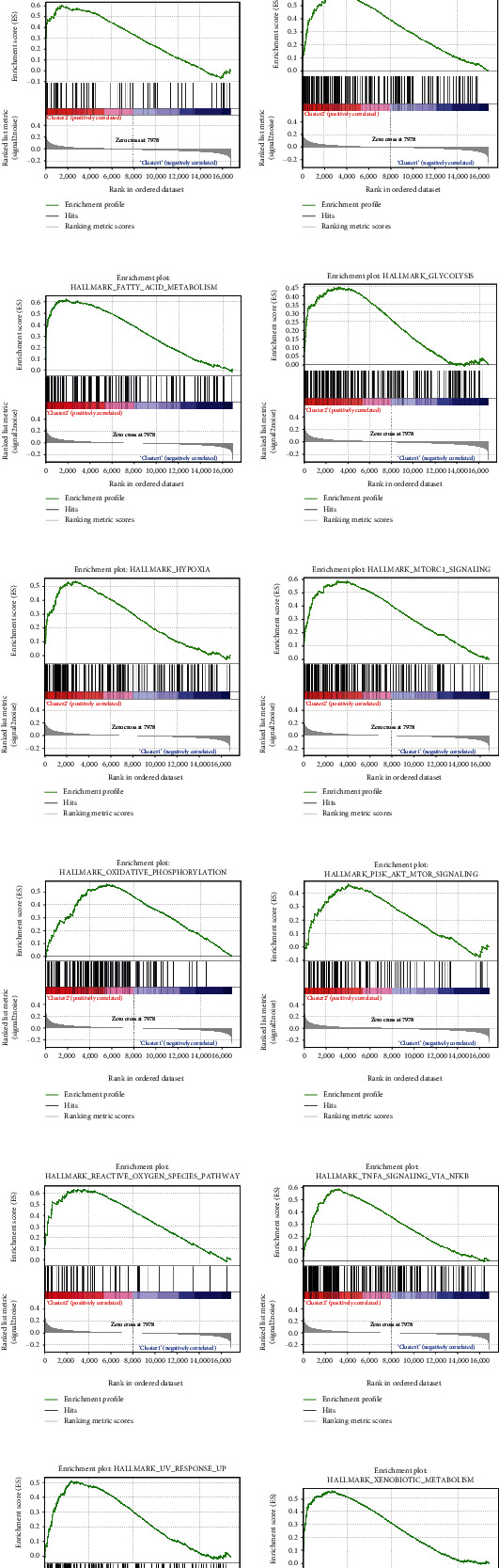
GSEA in adipogenesis subgroups. (a–n) Hallmark gene sets enriched in cluster 2 with a *p* value <0.05.

To explore the difference between other biological processes in two adipogenesis subgroups, GSEA was applied, and we could find that most genes were enriched in metabolic processes, such as fatty acid metabolism, oxidative phosphorylation, cholesterol homeostasis, and glycolysis. Notably, hypoxia, apoptosis, and complement were also significantly activated in cluster 2, which were reported to be associated with DPN pathogenesis [1, 31]. Besides, mTOC1, PI3K/AKT/mTOR, TNF-*α* signaling, and reactive oxygen species pathway were enriched in cluster 2 (Figures 4(a)–4(n), Table S5). These classical signal pathways mediated immune infiltration [32, 33], indicating that regulation of these signaling pathways may control the inflammatory response in DPN.

### 3.5. Differential Expression Analysis, Function Enrichment Analysis, and Hub Gene Selection

**Figure 5:**
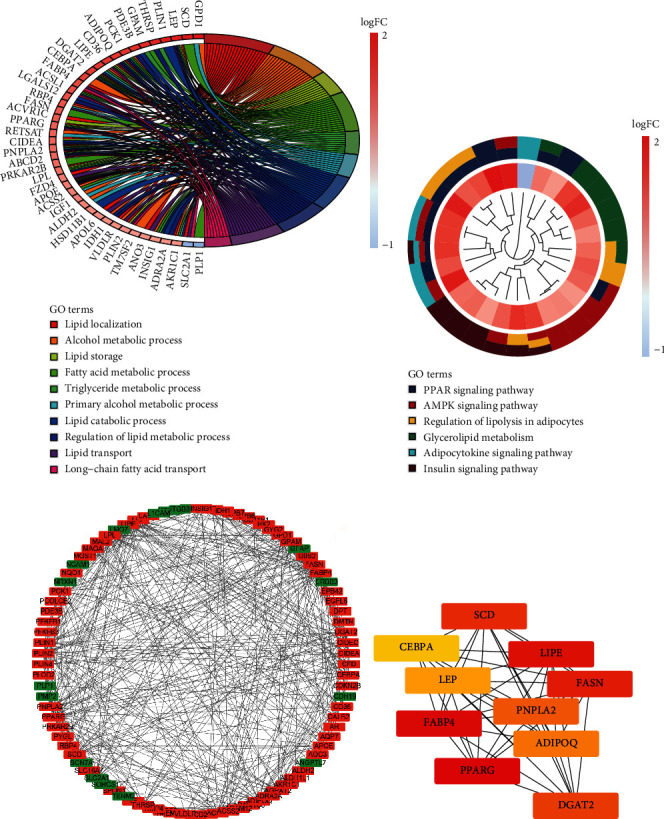
Differential expression analysis, function enrichment analysis, and hub gene selection. (a) A circular plot for GO enrichment analysis of the DEGs in two adipogenesis subgroups. (b) A clustering circular plot for KEGG analysis of the DEGs in two adipogenesis subgroups. (c) Protein-protein interaction network construction and analysis of differentially expressed genes. Red nodes represent upregulated genes, and green nodes represent downregulated genes. (d) Top 10 candidate genes with maximal clique centrality, including PPARG, FABP4, LIPE, FASN, SCD, DGAT2, PNPLA2, ADIPOQ, LEP, and CEBPA.

To gain more insight into molecular characteristics associated with adipogenesis subgroups, the “limma” package was utilized to discover DEGs in two adipogenesis subgroups. 131 DEGs were extracted, and most of them were upregulated in cluster 2 (Figure S5A, Table S6). GO analysis indicated that DEGs have mainly enriched lipid metabolism-related biological processes, including lipid storage, fatty acid metabolic process, and triglyceride metabolic process (Figure 5(a), Table S7). KEGG pathway enrichment analysis illustrated that more adipogenesis-related signaling pathways were enriched in cluster 2 (such as the PPAR signaling pathway, AMPK signaling pathway, and adipocytokine signaling pathway) (Figure 5(b), Table S8). In addition, we obtained interactive information among DEGs via the STRING online tool and established a prospective protein-protein interaction network. Red represented upregulation, and green represented downregulation (Figure 5(c)). As shown in Figure 5(d), we identified top 10 hub genes (PPARG, FABP4, LIPE, FASN, SCD, DGAT2, PNPLA2, ADIPOQ, LEP, and CEBPA) of the global PPI network according to the results of MCC algorithm using cytoHubba plugin.  Table 1 showed their full names and functions. These genes may perform as key genes to identify different adipogenesis subgroups.

### 3.6. Immune Correlation, TFs, and miRNA of Hub Genes

**Figure 6:**
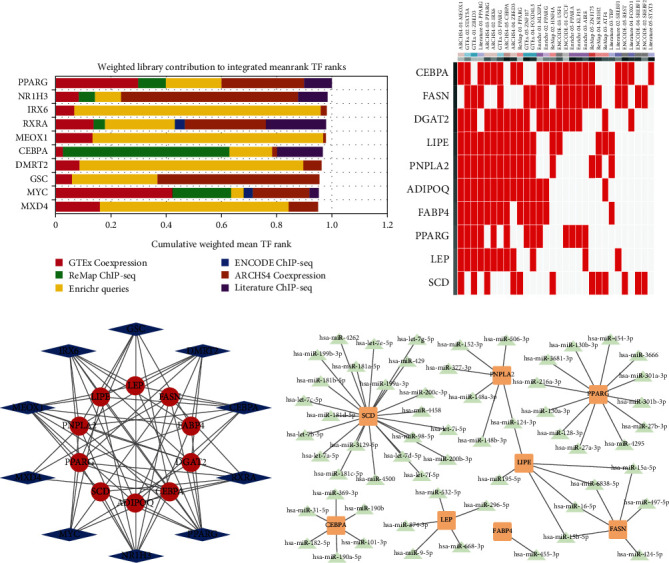
Predicted TFs of hub genes. (a) Top 10 predicted TFs for hub genes, including PPARG, NR1H3, IRX6, RXRA, MEOX1, CEBPA, DMRT2, GSC, MYC, and MXD4. (b) The heat map represents TF predicted by different databases (ENCODE, ReMap, GTEx, Enrichr, and ARCHS4). (c) PPI network between hub genes and the top ten predicted TF. (d) Interaction between hub genes and predicted miRNA.

To further investigate the relationship between hub genes and immune infiltration, correlation analysis was performed. It revealed that the expression of hub genes was remarkably associated with macrophage cell, Th1 cell, TIL cell, inflammation-promoting, parainflammation, T cell coinhibition, and T cell costimulation, which were also enriched in cluster 2 (Figure S5B). Then, we predicted the top 10 TFs that potentially regulated hub genes, including PPARG, NR1H3, IRX6, RXRA, MEOX1, CEBPA, DMRT2, GSC, MYC, and MXD4 (Figure 6(a)). The heat map represents TF predicted by different databases, including ENCODE, ReMap, GTEx, Enrichr, and ARCHS4 databases (Figure 6(b)). PPI network was used to present the interaction between hub genes and predicted top 10 TF (Figure 6(c)). What is more, we also predicted the miRNAs that may regulate hub genes that we found (Figure 6(d), Table 2).

### 3.7. Expression of Hub Genes in Sciatic Nerve Tissues

**Figure 7:**
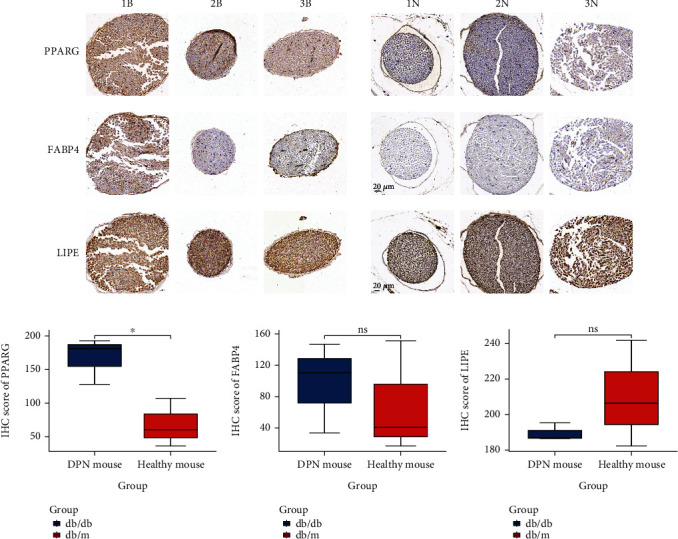
Expression of PPARG, FABP4, and LIPE in sciatic nerve tissues from db/db mice and db/m mice (Magnification ×400).

Using sciatic nerve tissues from DPN mouse and healthy mouse, we detected the expression of the top three hub genes (PPARG, FABP4, and LIPE) via IHC (Figure 7(a), Table S9). The results revealed that only the expressions of PPARG were considerably increased in sciatic nerve tissues from DPN mouse compared with the corresponding normal nerve tissues from healthy mouse (Figures 7(b)–7(d)). Taken together, our results suggested that PPARG may play an essential role in the diagnosis of DPN.

## 4. Discussion

Dyslipidemia is an important factor in DPN progression. Reduction of blood lipid and weight loss helps to delay the occurrence of DPN. More and more research suggested that adipogenesis can promote the development of DPN, but most of them focus on a single gene [2, 34, 35]. It is necessary to discover the expression patterns of multiple adipogenesis-related genes and their relationship with DPN. Since different genes often interact to jointly regulate the development of diseases, it is of great significance to explore the polygenic expression pattern of DPN [36]. In this study, 112 DPN patients were selected for unsupervised clustering and found that DPN patients could be divided into two significantly different subgroups according to the expression levels of DEARGs. DPN patients with high adipogenic levels had an increased level of immune infiltration and inflammatory response, indicating that disturbed lipid metabolism was associated with immune infiltration. Further, we screened the hub genes between two subgroups and predicted their potential TFs and miRNAs involved in the regulation. Some validations were performed to identify the expression of hub genes in the peripheral nerve. The results above may help us to further conduct mechanistic studies of the hub genes in DPN.

Excessive lipid synthesis often leads to chronic low-grade inflammation and immune infiltration, which may further cause other tissue damage. In pathological states such as obesity and metabolic excess, adipocytes often recruit more proinflammatory macrophages and other immune cells, and cytokines (such as TNF and IL-1) will significantly elevate to inhibit excessive adipogenesis [37]. DPN is also considered to be significantly associated with the peripheral immune response and inflammation. A previous study found that DPN animal models can overexpress TNF-*α* and IL-6, which were known as inflammatory factors [38]. Persistent neurogenic inflammation attracted innate and adaptive immune cells, especially macrophages [39]. There was a consensus that macrophages were the most important immune cell in DPN. Macrophages played an important role in innate immunity M1-like phenotype that can aggravate DPN by their excessive production of proteases, cytokines, and reactive oxygen species [40]. The infiltration of blood macrophages in the spinal cord may promote the development of painful neuropathy in diabetic patients [41]. Studies also showed that the inhibition of the release of M1 and macrophages into M2 macrophages could induce the gradual recovery of nerve conduction velocity, nerve blood flow, and axonal morphology in streptomycin-induced diabetic rats [42]. In our study, macrophages were highly enriched in cluster 2, which overexpressed adipogenesis and inflammatory-related genes. This may suggest that excessive lipid synthesis led to the recruitment of macrophages, which further mediates the damage of peripheral nerves in diabetes. Additionally, CD8+ T cells and CD4+ T cells also increased in cluster 2. Although CD8+ T cells mainly participated in adaptive immune, they have been reported to induce obvious apoptosis of Schwann cells [43]. A quantitative immunohistochemical study, which was carried out on 20 cases of DPN sural nerve biopsy specimens, had also found that diabetic nerve T cells infiltrated mainly CD8+ cells [44]. An imbalance of CD4+ T regulatory cells (Tregs) was also critical in the development of insulin resistance and diabetes [45]. Thus, although the cause-effect relationship between immune infiltration and DPN is uncertain, we tend to believe that some certain immune infiltrates lead to DPN. Anti-inflammatory therapy can reduce pain in DPN, while modulation of adipogenesis can moderate immune infiltration. Regulation of adipogenesis and immune infiltration may be the effective and reliable treatment of DPN.

To further explore the application of adipogenesis subgroups in DPN, we identified ten hub genes between two subgroups. Relevant studies had shown that these hub genes were associated with DPN and related metabolism. Peroxisome proliferator-activated receptor gamma (PPARG) is a regulator of adipocyte differentiation, which can express PPAR*γ* and is known to be important for ameliorating DPN. Besides, multiple drugs or small molecules could improve DPN by modulating the PPAR pathway [29, 46, 47]. Fatty acid binding protein 4 (FABP4) mainly participates in fatty acid uptake, transport, and metabolism. Antibody-mediated targeting of the hormone complex forms and improves metabolic outcomes, enhances cellular function, and maintains cellular integrity to prevent type 1 and type 2 diabetes [48]. Notably, FABP4 could regulate lipogenesis by downregulating PPAR*γ* and then have an affection on DPN [49]. Stearoyl-CoA desaturase (SCD) is an integral membrane protein located in the endoplasmic reticulum, involved in the synthesis of fatty acids, mainly oleic acid. Studies in transgenic mouse models had demonstrated an important role of SCD in regulating cellular processes, including lipid synthesis and oxidation, thermogenesis, hormone signaling, and inflammation [50]. Lipase E (LIPE) expresses a key enzyme for lipolysis, which hydrolyzes stored triglycerides into free fatty acids, and improves insulin resistance by participating in the regulation of fat metabolism [51]. Metformin and resveratrol inhibited PKA/LIPE activation, thereby inhibiting adipolysis, reducing FFA influx and DAG accumulation, and improving insulin signaling [52]. Interleukin 4 (IL-4) could also inhibit lipogenesis and promote adipolysis to reduce lipid deposition by enhancing the activity of LIPE [53]. Fatty acid synthase (FASN) is a multifunctional protein that mainly functions to catalyze the synthesis of palmitate growth chain saturated fatty acid by acetyl-CoA and malonyl-CoA in the presence of NADPH, which was closely related to immune response. Several studies had shown that miRNA could inhibit inflammation by targeting FASN to improve the progression of diabetes complications [54]. Interestingly, as the core gene of adipogenic subgroups, their high expression is also related to the high abundance of immune cells, which may have relationship in their participation in immune-related mechanisms. PPAR*γ* is a key factor in regulating, at least some aspects of macrophage lipid metabolism. The study had shown that Brd4 binds to the promoter and enhancer of GdF3 to promote PPAR*γ*-dependent expression of GdF3 in macrophages and modulated lipid metabolism and diet-induced obesity [55]. In DCs, T cells, and other immune cells, researchers detected upregulation of lipid metabolism and transport-related genes upon PPAR*γ* ligand treatment and found that upregulation of these genes could be blocked by PPAR*γ*-specific antagonist suggesting a PPAR*γ*-dependent regulation [56]. The role of FABP in regulating immunity had been confirmed in many diseases. Macrophage-FABP4 promoted the crosstalk between macrophages and neutrophils by regulating the production of CXCL1 in macrophages, thus playing a new role in the defense of lung hosts against Pseudomonas aeruginosa infection [57]. In addition, both FABP4 and FABP5 were involved in maintaining T lymphocyte homeostasis by regulating cytokine production, which might be regulated by cellular fatty acid-mediated signaling in thymic epithelial cells [58]. SCD1 expressed in cancer cells and immune cells could cause immune resistance, and its inhibition enhanced the therapeutic effect of antitumor T cells and anti-PD-1 antibodies [59]. LPS-induced macrophages were characterized by enhanced endogenous fatty acid synthesis and downregulation of the proinflammatory response by inhibition of fatty acid synthase (FASN). Further research showed that metformin could inhibit the elevation of FASN and the proinflammatory activation in macrophages [60]. There was evidence that Toll-like receptor-mediated inflammation required FASN-dependent MYD88 palmitoylation [30]. Another study showed that the link between FASN and cholesterol synthesis was required for TLR signal transduction and proinflammatory macrophage activation [61]. Meanwhile, overexpression of FASN could lead to the downregulation of immune-related genes [62]. These hub genes play a crucial role in the progression of diabetes complications by regulating fat metabolism and immune infiltration and regulating insulin resistance. By analyzing the correlation between immune infiltration and the screened hub genes, we also found that expression of hub genes was positively correlated with the enrichment levels of immune cells and immune processes, which suggested that these hub genes may represent the characteristics of cluster 2.

Although our findings seem encouraging, this study is still insufficient. First, the results of immune infiltration in adipogenesis subgroups were analyzed by ssGSEA, which uses a gene signature-based method to estimate the relative abundance of immune cells. Tissue-based flow cytometry may provide more accurate results. Besides, we applied immunohistochemistry to verify the expression of five hub genes in the DPN sciatic tissues, and the intensive mechanistic studies of these genes still need to be further improved in future studies. This will be the direction of our research in the future.

## 5. Conclusion

Briefly, our integrated analysis of adipogenesis subgroups revealed the relationship between adipogenesis, immune cell, and inflammatory process and identified PPARG, FABP4, LIPE, FASN, SCD, DGAT2, PNPLA2, ADIPOQ, LEP, and CEBPA as auxiliary diagnostic indicators for adipogenesis subgroups in DPN. These findings emphasized the importance of adipogenesis and would provide a new perspective for immunotherapy of DPN patients. However, future studies with larger samples and clinical information using flow cytometry analysis are warranted to validate these findings.

## Figures and Tables

**Table 1:** Hub genes and their functions.

Gene symbol	Full name	Function
PPARG	Peroxisome proliferator-activated receptor gamma	The protein encoded by this gene is PPAR*γ*, which is a regulator of adipocyte differentiation.

FABP4	Fatty acid binding protein 4	FABP4 encodes the fatty acid binding protein binding long-chain fatty acids and other hydrophobic ligands. It is thought that FABPs' roles include fatty acid uptake, transport, and metabolism.

LIPE	Lipase E, hormone sensitive type	The long form is expressed in steroidogenic tissues such as testis, where it converts cholesteryl esters to free cholesterol for steroid hormone production. The short form is expressed in adipose tissue, among others, where it hydrolyzes stored triglycerides to free fatty acids.

FASN	Fatty acid synthase	Its main function is to catalyze the synthesis of palmitate from acetyl-CoA and malonyl-CoA, in the presence of NADPH, into long-chain saturated fatty acids.

SCD	Stearoyl-CoA desaturase	This gene encodes an enzyme involved in fatty acid biosynthesis, primarily the synthesis of oleic acid.

DGAT2	Diacylglycerol O-acyltransferase 2	It catalyzes the final reaction in the synthesis of triglycerides in which diacylglycerol is covalently bound to long-chain fatty acyl-CoAs.

PNPLA2	Patatin-like phospholipase domain-containing 2	This gene encodes an enzyme which catalyzes the first step in the hydrolysis of triglycerides in adipose tissue. Mutations in this gene are associated with neutral lipid storage disease with myopathy.

ADIPOQ	Adiponectin, C1Q, and collagen domain containing	The encoded protein circulates in the plasma and is involved with metabolic and hormonal processes. Mutations in this gene are associated with adiponectin deficiency.

LEP	Leptin	This gene encodes a protein that is secreted by white adipocytes into the circulation and plays a major role in the regulation of energy homeostasis.

CEBPA	CCAAT enhancer-binding protein alpha	Activity of this protein can modulate the expression of genes involved in cell cycle regulation as well as in body weight homeostasis.

**Table 2:** Predicted miRNA.

Gene symbol	miRNA
PPARG	Hsa-miR-27a-3p; hsa-miR-216a-3p; hsa-miR-27b-3p; hsa-miR-128-3p; hsa-miR-130a-3p; hsa-miR-301a-3p; hsa-miR-130b-3p; hsa-miR-454-3p; hsa-miR-301b-3p; hsa-miR-4295; hsa-miR-3666; hsa-miR-3681-3p

FABP4	Hsa-miR-455-3p

LIPE	Hsa-miR-15a-5p; hsa-miR-16-5p; hsa-miR-15b-5p; hsa-miR-124-3p; hsa-miR-195-5p; hsa-miR-6838-5p

FASN	Hsa-miR-15a-5p; hsa-miR-16-5p; hsa-miR-15b-5p; hsa-miR-195-5p; hsa-miR-424-5p; hsa-miR-497-5p; hsa-miR-6838-5p

SCD	Hsa-let-7a-5p; hsa-let-7b-5p; hsa-let-7c-5p; hsa-let-7d-5p; hsa-let-7e-5p; hsa-let-7f-5p; hsa-miR-98-5p; hsa-miR-199a-3p; hsa-miR-181a-5p; hsa-miR-181b-5p; hsa-miR-181c-5p; hsa-miR-199b-3p; hsa-miR-200b-3p; hsa-let-7 g-5p; hsa-let-7i-5p; hsa-miR-124-3p; hsa-miR-200c-3p; hsa-miR-429; hsa-miR-181d-5p; hsa-miR-3129-5p; hsa-miR-4262; hsa-miR-4458; hsa-miR-4500

DGAT2	—

PNPLA2	Hsa-miR-148a-3p; hsa-miR-124-3p; hsa-miR-152-3p; hsa-miR-377-3p; hsa-miR-148b-3p; hsa-miR-506-3p

ADIPOQ	—

LEP	Hsa-miR-9-5p; hsa-miR-296-5p; hsa-miR-532-5p; hsa-miR-668-3p; hsa-miR-874-3p

CEBPA	Hsa-miR-31-5p; hsa-miR-101-3p; hsa-miR-182-5p; hsa-miR-124-3p; hsa-miR-190a-5p; hsa-miR-369-3p; hsa-miR-190b

## Data Availability

All the datasets we used in this study can be found in Gene Expression Omnibus (GEO, https://www.ncbi.nlm.nih.gov/geo/). All details of these data sets were shown in supplementary table.

## Supplementary Materials

Supplementary 1 Supplementary Figure 1: the entire analytical process of our study (created with http://BioRender.com). Supplementary Figure 2: boxplot of samples from GSE24290 and GSE148059 before (a) and after (b) removing batch effect. Supplementary Figure 3: consensus clustering. (a–g) Consensus matrix heatmaps for *k* = 3 − 9. (h, i) Consensus CDF curve and delta area curve of consensus clustering analysis. *k* = 2 was regarded as appropriate. Supplementary Figure 4: heat map of inflammation-related genes in cluster 1 and cluster 2. Supplementary Figure 5: (a) heat map of differentially expressed genes between cluster 1 and cluster 2. Red represented upregulation, and green represented downregulation in cluster 2. (b) correlation analysis of hub genes and immune cell/immune process. (^∗^*p* < 0.05, ^∗∗^*p* < 0.01, and ^∗∗∗^*p* < 0.001).

Supplementary 2 See Table S1-S9 for raw data. Table S1: differential expression analysis of GSE95849. Table S2: differentially expressed adipogenesis-related genes. Table S3: merged dataset with limma package. Table S4: results of ssGSEA. Table S5: results of GSEA. Table S6: differential expression analysis between cluster 1 and cluster 2. Table S7: GO analysis. Table S8: KEGG analysis. Table S9: IHC assay.
