# Supplementary material for: Identification of Adipogenesis Subgroups and Immune Infiltration Characteristics in Diabetic Peripheral Neuropathy
Source: J Immunol Res. 2023 Jan 19;2023:3673094. doi: 10.1155/2023/3673094 (PMC9893521; doi:10.1155/2023/3673094)
Supplement: Supplementary 1 — Supplementary Figure 1: the entire analytical process of our study (created with http://BioRender.com). Supplementary Figure 2: boxplot of samples from GSE24290 and GSE148059 before (a) and after (b) removing batch effect. Supplementary Figure 3: consensus clustering. (a–g) Consensus matrix heatmaps for k = 3 − 9. (h, i) Consensus CDF curve and delta area curve of consensus clustering analysis. k = 2 was regarded as appropriate. Supplementary Figure 4: heat map of inflammation-related genes in cluster 1 and cluster 2. Supplementary Figure 5: (a) heat map of differentially expressed genes between cluster 1 and cluster 2. Red represented upregulation, and green represented downregulation in cluster 2. (b) correlation analysis of hub genes and immune cell/immune process. (∗p < 0.05, ∗∗p < 0.01, and ∗∗∗p < 0.001). [file 3673094.f1.docx]

Supplementary Figure


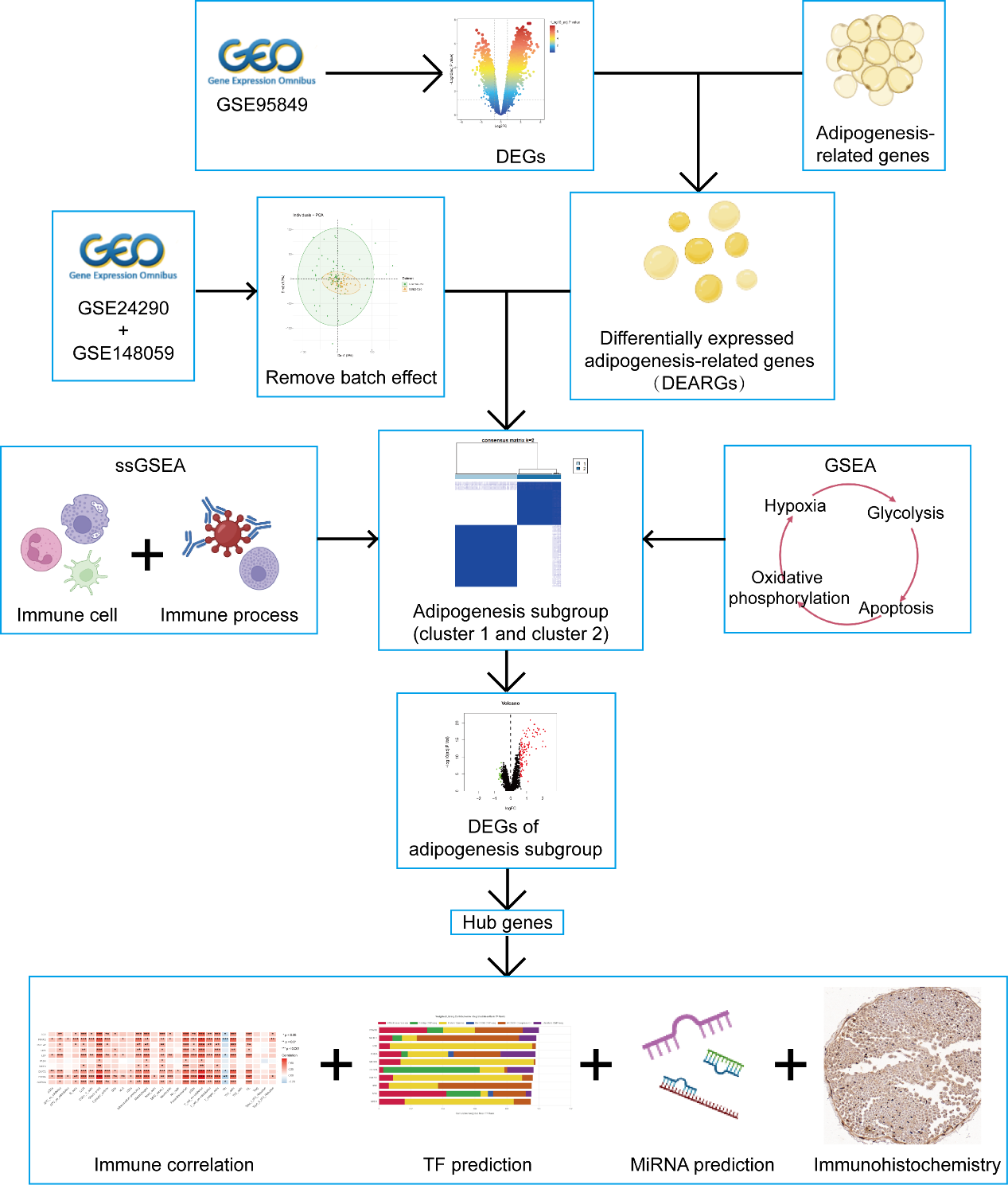


**Supplementary Figure 1** The entire analytical process of our study. (Created with BioRender.com)


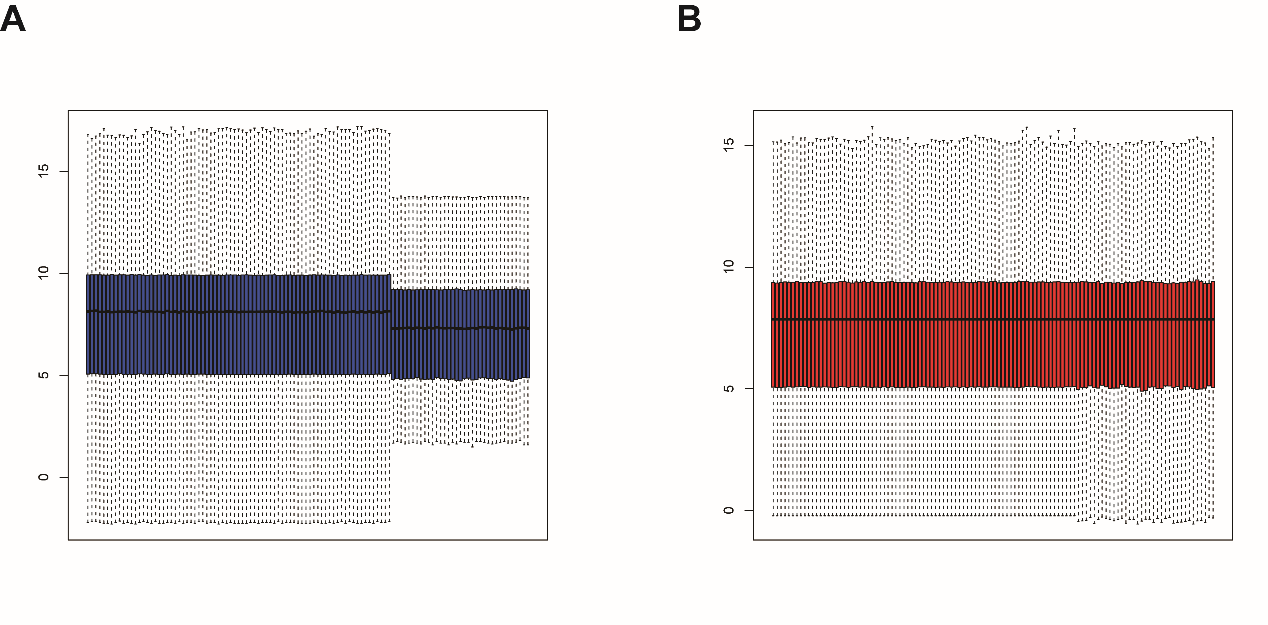


**Supplementary Figure 2** Boxplot of samples from GSE24290 and GSE148059 before (A) and after (B) removing batch effect.


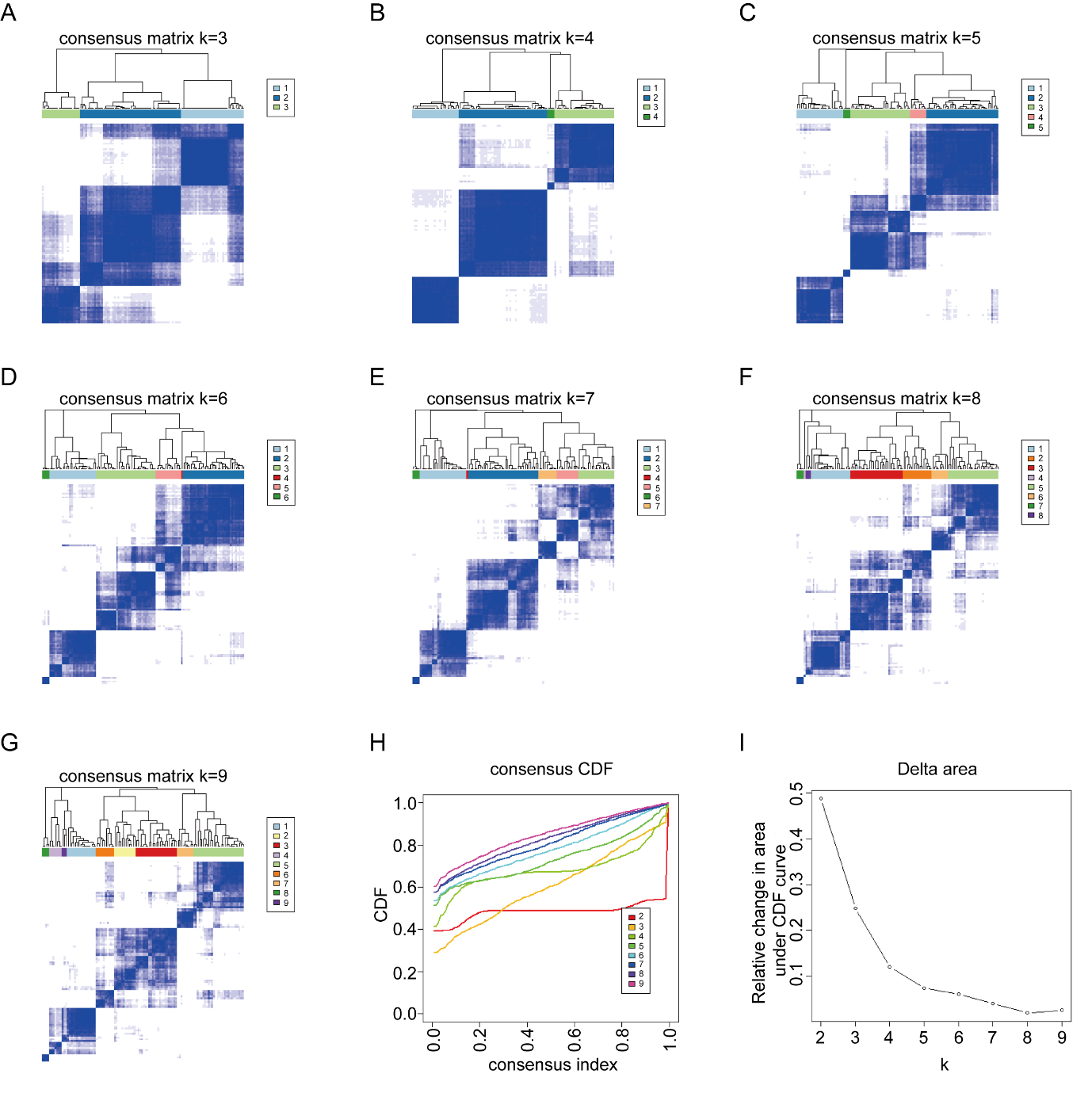


**Supplementary Figure 3** Consensus clustering (A-G) Consensus matrix heatmaps for k=3-9. (H-I) Consensus CDF curve and Delta area curve of consensus clustering analysis. K=2 was regarded as appropriate.


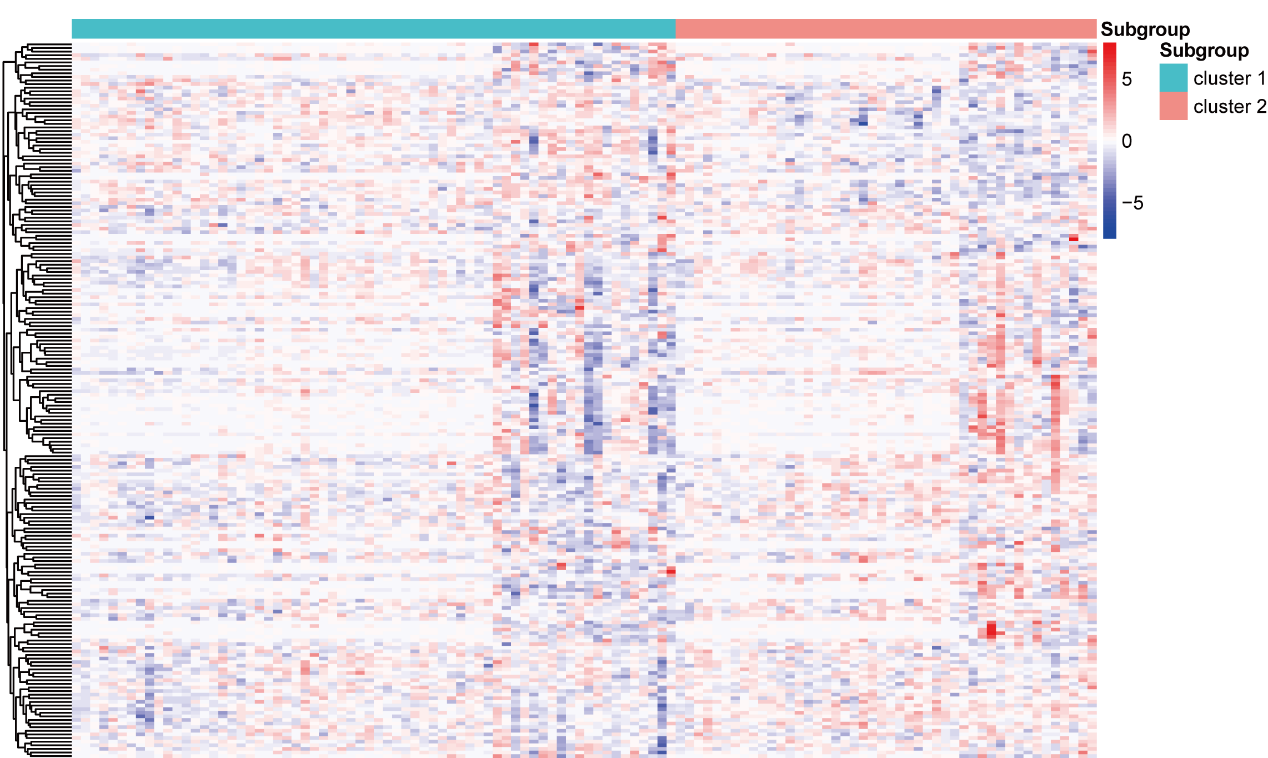


**Supplementary Figure 4** Heatmap of inflammation- related genes in cluster 1 and cluster 2


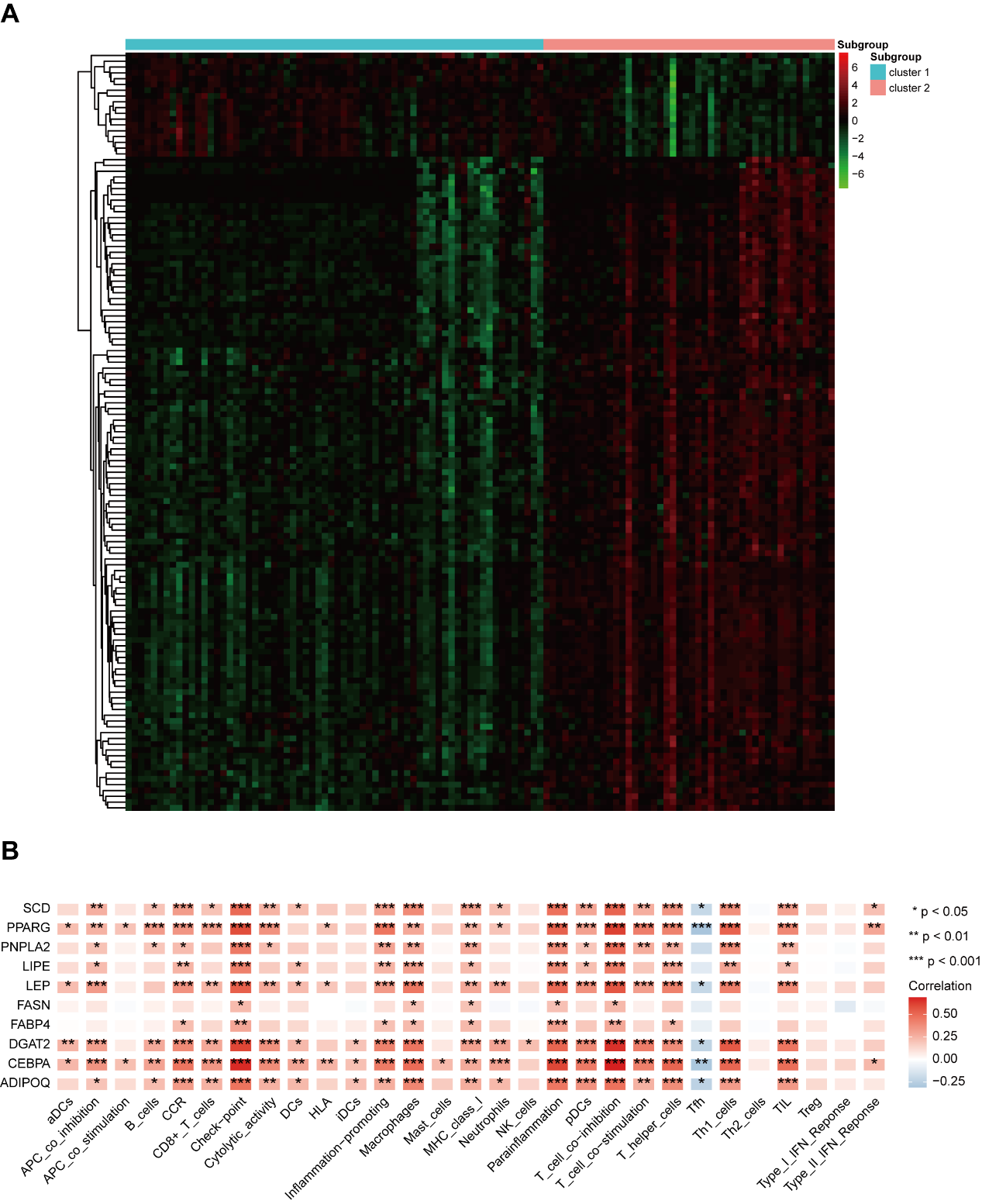


**Supplementary Figure 5** (A) Heatmap of differentially expressed genes between cluster 1 and cluster 2. Red represented up-regulation and green represented down-regulation in cluster 2. (B) correlation analysis of hub genes and immune cell/immune process. (**p* < 0.05, ***p* < 0.01, ****p* < 0.001).
